# Electron tomography imaging methods with diffraction contrast for materials research

**DOI:** 10.1093/jmicro/dfaa002

**Published:** 2020-03-02

**Authors:** Satoshi Hata, Hiromitsu Furukawa, Takashi Gondo, Daisuke Hirakami, Noritaka Horii, Ken-Ichi Ikeda, Katsumi Kawamoto, Kosuke Kimura, Syo Matsumura, Masatoshi Mitsuhara, Hiroya Miyazaki, Shinsuke Miyazaki, Mitsu Mitsuhiro Murayama, Hideharu Nakashima, Hikaru Saito, Masashi Sakamoto, Shigeto Yamasaki

**Affiliations:** 1 Department of Advanced Materials Science, Kyushu University, Fukuoka 816-8580, Japan; 2 The Ultramicroscopy Research Center, Kyushu University, Fukuoka 819-0395, Japan; 3 TEMography Division, System in Frontier Inc., Tachikawa-shi, Tokyo 190-0012, Japan; 4 Research Laboratory, Mel-Build Corporation, Fukuoka 819-0025, Japan; 5 Steel Research Laboratories, Nippon Steel Corporation, Chiba 293-8511, Japan; 6 Division of Materials Science and Engineering, Faculty of Engineering, Hokkaido University, Hokkaido 060-8628, Japan; 7 Morphological Research Laboratory, Toray Research Center, Inc., Shiga 520-8567, Japan; 8 Department of Applied Quantum Physics and Nuclear Engineering, Kyushu University, Fukuoka 819-0395, Japan; 9 Analytical Instruments, Materials and Structural Analysis, Thermo Fisher Scientific, Shinagawa-ku, Tokyo 140-0002, Japan; 10 Department of Materials Science and Engineering, Virginia Tech, Blacksburg, VA 24061, USA; 11 Energy and Environmental Directorate, Pacific Northwest National Laboratory, WA 99352, USA; 12 Institute for Materials Chemistry and Engineering, Kyushu University, Fukuoka 816-8580, Japan

**Keywords:** electron tomography, three-dimensional (3D), diffraction contrast, domain structure, dislocation, specimen holder

## Abstract

Transmission electron microscopy (TEM) and scanning transmission electron microscopy (STEM) enable the visualization of three-dimensional (3D) microstructures ranging from atomic to micrometer scales using 3D reconstruction techniques based on computed tomography algorithms. This 3D microscopy method is called electron tomography (ET) and has been utilized in the fields of materials science and engineering for more than two decades. Although atomic resolution is one of the current topics in ET research, the development and deployment of intermediate-resolution (non-atomic-resolution) ET imaging methods have garnered considerable attention from researchers. This research trend is probably not irrelevant due to the fact that the spatial resolution and functionality of 3D imaging methods of scanning electron microscopy (SEM) and X-ray microscopy have come to overlap with those of ET. In other words, there may be multiple ways to carry out 3D visualization using different microscopy methods for nanometer-scale objects in materials. From the above standpoint, this review paper aims to (i) describe the current status and issues of intermediate-resolution ET with regard to enhancing the effectiveness of TEM/STEM imaging and (ii) discuss promising applications of state-of-the-art intermediate-resolution ET for materials research with a particular focus on diffraction contrast ET for crystalline microstructures (superlattice domains and dislocations) including a demonstration of *in situ* dislocation tomography.

## Introduction

Electron tomography (ET) is a three-dimensional (3D) imaging method based on transmission electron microscopy (TEM) and scanning transmission electron microscopy (STEM). ET reconstructs 3D nanoscale objects observed in a TEM/STEM field of view in a computer and enables the observation and analysis of the 3D morphology of the reconstructed objects. Such ET nanostructural characterizations have now spread into the research field of materials science and engineering (MSE) as well as that of biological and medical sciences.

Because modern TEM/STEM apparatuses are capable of various types of nanostructural imaging, ET observations can be performed using multiple imaging methods. The incoherent annular dark-field (ADF) STEM imaging method in which the inner collection angle of an annular detector is normally larger than 40 mrad is a standard imaging method in ET for materials research [1–10] and is now capable of achieving atomic-scale 3D spatial resolutions [11–19]. Spectroscopic ET methods with energy-dispersive X-ray spectroscopy (EDXS) [20–27] or electron energy-loss spectroscopy (EELS) [20, 28–36] have become promising methods for visualizing not only 3D morphologies but also various properties of objects in three dimensions. The use of these advanced ET methods is closely related with recent significant developments in 3D reconstruction methods that can reduce artifacts caused by various kinds of missing information in tilt-series projection data sets. Attempts are being made to make high-quality advanced ET data sets available to facilitate further developments in 3D reconstruction methods [37]. Modern image acquisition devices such as direct detection camera systems [38, 39] are accelerating the development of *in situ* TEM tomography that visualizes the 4D (space and time) dynamic behavior of materials [39–43]. Applications of STEM to *in situ* ET have also been reported recently [19, 44].

### Fundamental issues of ET imaging methods

When performing ET investigations, it should be taken into account that there are fundamental issues with ET imaging methods, as described below.

The first issue is the so-called missing wedge artifact, which appears in the 3D volume when reconstructed using ET. When the specimen for ET observation is a thin foil, it is generally challenging to acquire the tilt-series data sets of TEM/STEM images in the high specimen-tilt angular range, especially ±70–90°. Thus, information from that angular range is not available in the tilt-series data sets, and the missing information of 2D projections at such high specimen-tilt angles severely degrades the spatial resolution of ET along the direction of specimen thickness [4, 45–48].

**Fig. 1:**
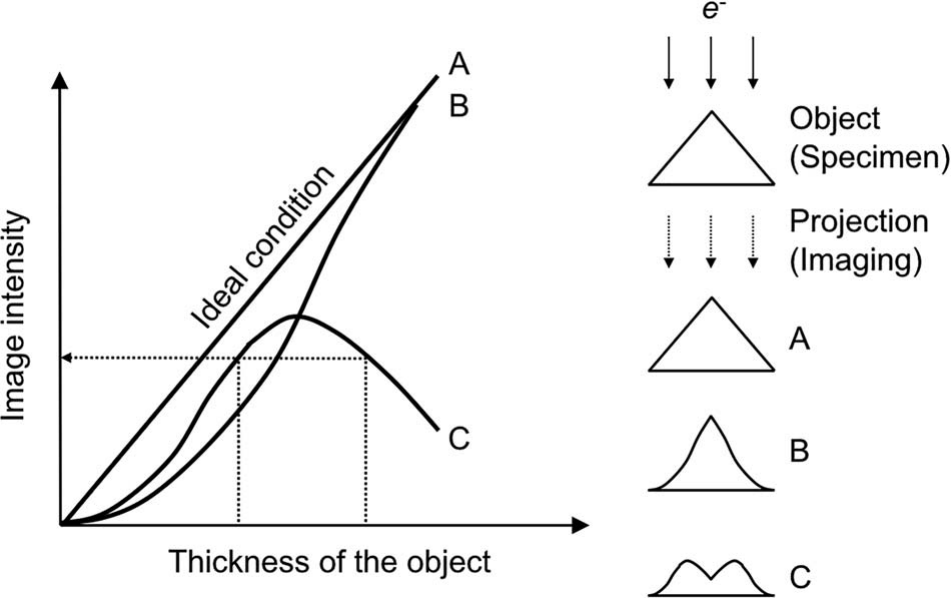
Explanation of the projection requirement [49]. Case A: perfect satisfaction of the projection requirement: the image intensity is proportional to the thickness of the object. Case B: sufficient satisfaction of the projection requirement: the image intensity is a monotonic function of the thickness. Case C: violation of the projection requirement: the image intensity is not a monotonic function of the thickness.

The second issue with ET is the violation of the projection requirement. Figure 1 [49] explains the projection requirement, i.e. the TEM/STEM image intensity of the object of interest must be a monotonic function of the projected physical properties of the object, namely, density and thickness, in order to reconstruct a reliable 3D volume of the object using ET. There are several potential causes for the projection requirement violation. One of the commonly observed is the absorption of incident electrons while traveling through a specimen. For example, when a thin foil specimen is used, the penetration length of the incident electrons increases with increasing the specimen-tilt angle, which is expressed as *L* = *t*/cos*θ*, where *L* is the penetration length, *t* is the specimen thickness and *θ* is the specimen-tilt angle. Now, let us consider a conventional incoherent ADF-STEM tomography imaging with a thin foil specimen. One would experience that the image intensity of tilt-series images is either saturated or dropped as the specimen-tilt angle becoming larger, normally |*θ*| > 60°. This appears to be a violation of the projection requirement, because the monotonic increment of *L*, which is equivalent to the effective specimen thickness, should lead a continuous increment of the image intensity. However, this can be understood that an increase in *L* more than a particular level for the large specimen-tilt angles indeed reduces the total number of electrons penetrated the specimen. As a result, the monotonic relationship between the image intensity and the specimen density/thickness is no longer maintained. Furthermore, if there is a significant reduction in the total number of transmitted electrons at high specimen-tilt angles, the acquired images at high specimen-tilt angles hardly contribute to the 3D image reconstruction [50]. This phenomenon also causes missing wedge artifacts.

The violation of the projection requirement also comes from dynamical diffraction contrasts, such as bend contours, thickness fringes and strain contrast. These kinds of dynamical diffraction contrasts often violate the projection requirement in a tilt-series data set. STEM also exhibits a similar diffraction contrast as compared to TEM. Nevertheless, the convergent beam illumination in STEM effectively weakens the dynamical diffraction contrast [51–68]. Thus, STEM is becoming a standard imaging mode for ET.

### Is ET the most suitable 3D imaging method for bulk materials?

There are several solutions for the fundamental issues of ET described above. For the projection requirement assessment, Yamasaki *et al.* [69, 70] proposed an empirical model to evaluate the maximal specimen thickness and suitable imaging conditions for ET using BF-TEM, such as the acceleration voltage and the objective aperture size, by measuring the incident electron transmittance through the specimen. Optimizing the specimen shape is an essential solution for achieving reliable ET observations of bulk materials. If a bulk material is shaped into a nanosized rod without losing the objects to be observed by ET, one can acquire ET data free from missing wedge artifacts and projection requirement violation [46]. Appropriate selection or development of image processing on a tilt-series data set and a mathematical algorithm for 3D volume reconstruction are essential for a reliable reconstruction of the 3D shape and/or density of an object. For example, noise reduction and background subtraction applying to a tilt-series data set are fundamental techniques that can contribute to improved satisfaction of the projection requirement [64, 71]. As far as the image intensity of a tilt-series data set that satisfies the projection requirement, when the tilt-series images are composed of a few components, in other words, images are being ‘sparse’ (e.g. metal nanoparticles and a supporting carbon film), discrete and/or compressed sensing approaches are so useful to reduce the missing wedge artifacts in 3D volume reconstruction [72–80].

The other solution for the fundamental issues of ET is the use of a different 3D visualization method other than ET, such as serial sectioning using scanning electron microscopy (SEM) in combination with focused ion beam (FIB) milling [81–85] or 3D X-ray microscopy [86–91]. These 3D imaging methods are free from missing wedge artifacts and are capable of reconstructing a significantly larger volume than is possible with typical ET. For example, transmission synchrotron X-ray tomographic microscopy was used to visualize the 3D microstructural evolution of sub-μm size precipitates in Al alloys [90]. Recently, these 3D imaging methods, namely, ET, SEM/FIB serial sectioning and X-ray microscopy, have shown partial overlap in feasible spatial resolution ranges of each other. In other words, ET may not necessarily be the most suitable imaging method for visualizing 3D objects with sizes in the ranges of 5–500 nm. In the next section, we describe the current status of typical ET methods with intermediate resolutions (non-atomic resolution) in materials research to clarify the prospects of ET.

## Review of ET imaging methods with intermediate resolutions

### Electron holographic tomography

A combination of electron holography with ET is capable of visualizing nanoscale electromagnetic fields in 3D. In the 1990s, Tonomura and his co-workers succeeded in visualizing not only electric potential fields but also magnetic vector fields of sub-μm size particles, which is recognized as the earliest application of ET to magnetic specimens [92]. In the 2000s, holographic ET techniques were applied to visualize the electric potential fields in semiconducting devices [93]. More recently, high-resolution 3D magnetic vector field visualization was carried out with holographic ET using aberration-corrected field-emission high-voltage TEM, which is a highly unique experimental strategy for visualizing 3D magnetic properties at a nanometer scale [94].

### STEM-EDX tomography

At the beginning of the 2000s, the combination of EDXS with STEM tomography was proposed as an effective approach for 3D materials characterization [1, 20]. Because the characteristic X-ray counts measured by STEM-EDXS are less susceptible to the electron diffraction phenomena in a crystal when compared with the image intensity in TEM, STEM-EDX tomography has been regarded as one of the promising ET methods together with incoherent ADF-STEM tomography [20–25]. Thanks to the development of large-area silicon drift detectors (SDDs) for EDXS [22–27] and tomographic reconstruction algorithms that work well with poor signal-to-noise ratio and reduce the number of projection images in a tilt-series data set [72–80], STEM-EDX tomography has received considerable attention as a new analytical electron microscopy function [36]. In a TEM/STEM, an EDX detector is usually located above the specimen (at the incident beam side). Thus, it should be noted that X-ray signals measured in the EDX detector are not merely equivalent to the transmitted electron signals measured below the specimen (at the transmitted beam side). In other words, one has to consider first the following: X-ray intensity maps acquired by STEM-EDXS are, in principle, inappropriate for tomographic 3D reconstruction. Therefore, it is recommended to use sufficiently thin specimens for EDX tomography to facilitate the detection of X-rays emitted from the bottom part of the specimen.

However, STEM-EDX tomography is recently starting to get more acceptance as a promising method than before because of another noteworthy feature: the possibility of reducing missing wedge artifacts due to SDDs equipped with a large X-ray detection area and an optimized detector design to enhance X-ray detection efficiency [22–27]. For example, in the case of an EDXS system composed of dual or quadruple SDDs, reducing the X-ray counts in one SDD by specimen tilt is compensated by increasing the X-ray counts in the other SDDs. Furthermore, when the specimen-tilt angle becomes high, those multiple SDDs measure the X-rays emitted from both sides of the foil specimen, resulting in increased X-ray counts in the tilt-series elemental maps acquired at high specimen-tilt angles. Such high X-ray counts in the elemental maps enhance their contribution to the subsequent 3D reconstruction process and finally improve the resolution power of the resultant 3D elemental maps. Theoretical calculations regarding the entire tomographic EDXS measurement process [25] and the iterative X-ray absorption correction [24] for quantitative STEM-EDX tomography have also been reported.

### Diffraction contrast ET

Diffraction contrast imaging is a fundamental imaging method of TEM/STEM with intermediate resolutions. Although incoherent imaging methods using ADF-STEM and STEM-EDXS are now operable and informative with regard to characterization of materials, they are not alternatives of diffraction contrast imaging methods in TEM/STEM. For example, TEM/STEM observations of the following microstructures use diffraction contrast: polycrystalline grains; crystal defects such as dislocations, stacking faults and twins; and orientation variants of non-cubic compound phases. Early diffraction contrast ET attempted to visualize the 3D shapes and distribution of an orientation variant of a coherently precipitated Ni_4_Mo tetragonal superlattice phase in Ni–Mo alloy [95], and the dislocation networks in epitaxially grown GaN films [71, 96]. Later, a full 3D visualization of the polycrystalline grain structure in Al was reported [97]. The following subsections describe the typical applications of diffraction contrast ET and the relevant hardware developments that have influenced other imaging methods.

#### Diffraction contrast ET applied to 3D crystalline objects with a superlattice structure

Multiple scattering processes in electron diffraction in crystals result in non-linear diffraction intensities with respect to crystal thickness. Therefore, diffraction contrast TEM/STEM is generally regarded as a non-suitable imaging method for ET. Nevertheless, diffraction contrast ET uses quasi-kinematical parts of electron diffraction intensities appearing in limited crystal thickness ranges and diffraction conditions. Here, the basic concept of diffraction contrast ET is explained in detail using the Ni_4_Mo compound phase.

**Fig. 2:**
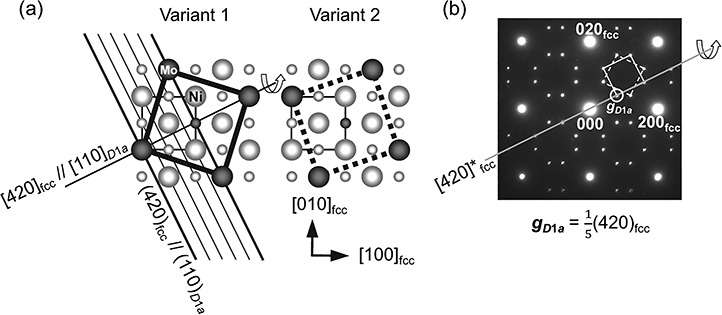
A crystal structure of Ni_4_Mo (a) and a corresponding electron diffraction pattern (b) [99]. Unit cells in two of the six orientation variants, variant 1 and 2, are drawn in (a), and each of the two variants exhibits superlattice reflections at different locations, as indicated by the two open squares in (b).

The crystal structure of Ni_4_Mo (tetragonal, Strukturbericht symbol of *D*1*_a_*, space group of No. 87 *I*4/*m*, lattice parameters of *a* = 0.5720 nm and *c* = 0.3564 nm [98]; orientation relationships between the *D*1_a_ structure and the fundamental face-centered cubic (fcc) structure: *a* [100]_*D*1*a*_, *b*[010]_*D*1*a*_//<310>_fcc_; *c*[001]_*D*1*a*_ = < 001>_fcc_) is shown in Fig. 2(a) [99]. Mo atoms periodically substitute the Ni atoms, thereby forming the face-centered cubic structure. The distance between the Mo atom planes is five times the spacing of (420) planes in the Ni matrix. The diffraction pattern calculated under the kinematical (single scattering) approximation in Fig. 2(b) exhibits fundamental lattice reflections, such as *hkl* = 200 and 220, and superlattice reflections at *hkl* = $\frac{1}{5}$(420), $\frac{1}{5}$(260) and their equivalent positions, where all the reciprocal lattice indices are presented based on the fundamental fcc lattice, for simplicity. When the Ni_4_Mo ordered phase forms from a Ni–Mo solid solution alloy with a disordered fcc structure, six equivalent orientation variants of the Ni_4_Mo phase appear. Figure 2 illustrates two of the six variants that have the common *c*-axis coinciding with the [001] axis of the Ni matrix [99]. Because each variant exhibits superlattice reflections at different locations in the reciprocal lattice, the DF imaging by the superlattice reflection visualizes one of the six variants.

**Fig. 3:**
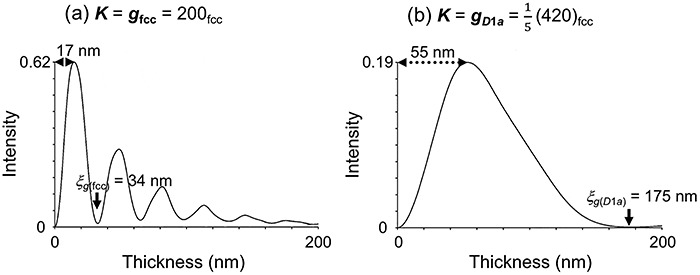
Calculated dynamical diffraction intensities in the Ni_4_Mo crystal as a function of crystal thickness [99]. (a) A fundamental lattice reflection, ***g***_**fcc**_ = 200_fcc_, under the exact Bragg condition, ***K*** = ***g***_**fcc**_, showing the effective extinction distance of *ξ*_***g*(fcc)**_ = 34 nm. (b) A superlattice reflection, ***g***_***D*1*a***_ = $\frac{1}{5}$(420)_fcc_, for ***K*** = ***g***_***D*1*a***_ showing *ξ*_***g*(*D*1*a*)**_ = 175 nm.

Figure 3 shows the calculated dynamical electron diffraction intensities of the fundamental lattice reflection, *hkl* = 200 (a), and the superlattice reflection, *hkl* = $\frac{1}{5}$(420) (b), as a function of Ni_4_Mo crystal thickness [99]. The extinction distances of the two reflections are 35 nm for (a) and 175 nm for (b) under the following conditions: an acceleration voltage of 200 kV and exact Bragg cases in systematic excitation conditions. The diffraction intensities increase monotonically up to 17 nm for (a) and 55 nm for (b). Based on this fact, if a Ni_4_Mo ordered alloy specimen is set on a TEM specimen holder in which the superlattice reflection at *hkl* = $\frac{1}{5}$(420) satisfies the Bragg condition on the specimen-tilt axis, one could acquire a tilt-series data set of dark-field (DF) TEM images while maintaining the Bragg condition. The acquired DF-TEM images may satisfy the projection requirement as long as the electron penetration lengths at the field of view are shorter than 55 nm, which is a fundamental concept of diffraction contrast ET for crystalline 3D objects with a superlattice structure.

**Fig. 4:**
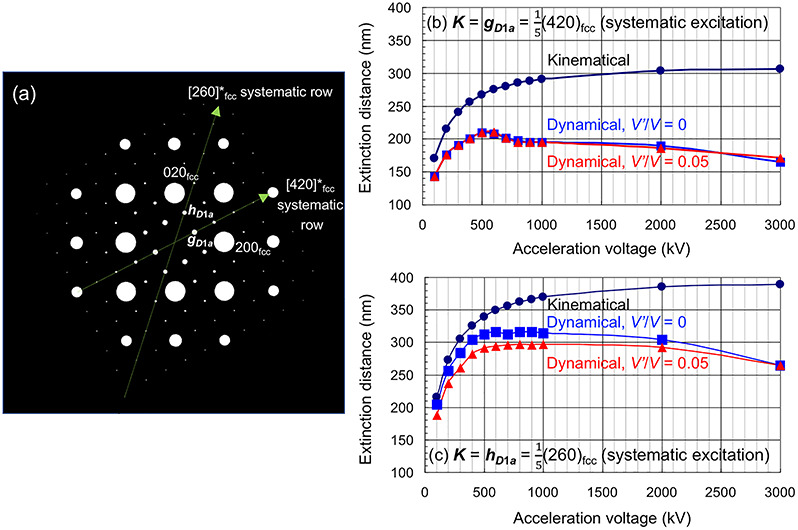
Calculated dynamical electron diffraction intensities of the fundamental lattice reflection, *hkl* = 200, and the superlattice reflection, *hkl* = $\frac{1}{5}$(420), as functions of the Ni_4_Mo crystal thickness. The calculations were performed using the software, JEMS [102], under the following conditions: acceleration voltage of 200 kV; the exact Bragg condition under the systematic excitation for each reflection; the ratio of inelastic scattering (absorption) and elastic scattering potential values for a diffracted wave ***g***, $\frac{V_{\boldsymbol{g}}^{\prime }}{V_{\boldsymbol{g}}}$ = 0.05 or 0.

Kimura *et al.* [95, 99–101] performed DF-TEM tomography observation and simulations on Ni_4_Mo alloys and investigated how the projection requirement is satisfied or violated depending on various experimental parameters. Based on their findings, they proposed the following essential points for obtaining reliable 3D reconstructed volumes of the Ni_4_Mo ordered phase by DF-TEM tomography: (i) selection of a higher acceleration voltage; (ii) selection of a low-index reflection; (iii) precise alignment to a particular Bragg condition; (iv) avoidance of low-index zone axis illumination conditions; (v) difficulties in 3D visualization of anti-phase domain boundaries; and (vi) use of an iteration-type 3D reconstruction algorithm. Here, point (i) is described in detail. The extinction distance for the superlattice reflection, ***g***_***D*1*a***_**=**$\frac{1}{5}$(420)_fcc_, increases with acceleration voltage, and the maximal crystal thickness that satisfies the projection requirement also increases, as shown in Fig. 4. In the model calculation, the extinction distance reached its maximal value (~210 nm) around 500 kV. Similar calculations for different reflections, such as for ***h***_***D*1*a***_**=**$\frac{1}{5}$(260)_fcc_, revealed that their extinction distances tend to saturate at 500–1500 kV and gradually decrease at higher voltages due to many-beam excitations.

**Fig. 5:**
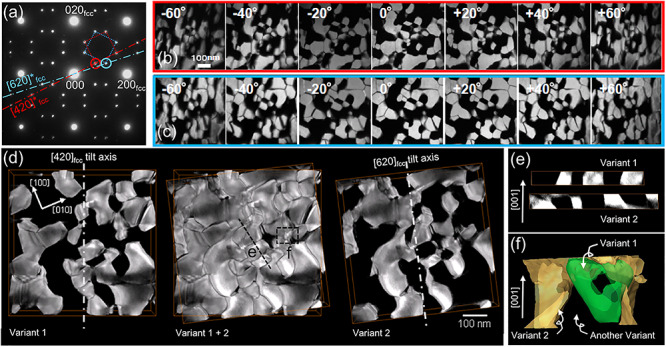
DF-TEM tomography observation of two orientation variants of tetragonal Ni_4_Mo domains in Ni–19.5 at.% Mo alloy [101]. (a) A [001] electron diffraction pattern explaining the two DF-TEM imaging conditions: ***g***_***D*1*a***_**=**$\frac{1}{5}$(420)_fcc_ excitation on the specimen-tilt axis for variant 1 and ***h***_***D*1*a***_**=**$\frac{1}{5}$(260)_fcc_ excitation for variant 2, (b) parts of DF-TEM tilt series for variant 1 (upper row) and variant 2 (lower row), (c) reconstructed 3D volumes of variant 1 (left), variant 2 (right) and their superposition (center), (d) 2D cross-sections of the reconstructed variant 1 and variant 2 along line A denoted in (c) and (e) a magnified 3D view of the superposition of variant 1 and variant 2 showing the existence of the other variant between them.

Figure 5 shows the DF-TEM tomography observation [101]. The specimen is a Ni_4_Mo (Ni–19.5 at % Mo) alloy, acquired by an ordering treatment at 1073 K for 24 h from the Ni solid solution state. In this alloy specimen, two of the six Ni_4_Mo variants with the common *c*-axis, namely, variant 1 and variant 2 (shown in Fig. 2), grow preferentially to form a two-variant structure. Here, the DF-TEM tomography observation aims to clarify how the two Ni_4_Mo variants fill a 3D space in the alloy. A thin foil specimen with a diameter of 3 mm was set on a specimen holder, in which the systematic row containing the superlattice reflection ***g***_***D*1*a***_**=**$\frac{1}{5}$(420)_fcc_ was oriented to the specimen-tilt axis, in order to observe variant 1, as shown in Fig. 5(a). The upper row in Fig. 5(b) shows parts of the DF-TEM tilt-series for variant 1 acquired under the following conditions: an electron microscope JEM-3200FSK; an acceleration voltage of 300 kV; a specimen-tilt range from −60° to +60°; a specimen-tilt increment of 2°; and a diffraction alignment by incident beam tilt to satisfy the Bragg condition for ***g*** in the systematic excitation at each specimen-tilt angle. After the tilt-series data set acquisition for variant 1, the specimen was rotated several degrees so that the other superlattice reflection ***h***_***D*1*a***_**=**$\frac{1}{5}$(260)_fcc_ was then parallel to the specimen-tilt axis in order to observe variant 2, as shown in Fig. 5(a). The lower row in Fig. 5(b) shows parts of the DF-TEM tilt-series for variant 2 which was acquired in the same way as that for variant 1. Figure 5(c) shows the 3D reconstructed volumes of variant 1 and variant 2 separately obtained from the two DF-TEM tilt-series data sets. The reconstructed variant 1 and variant 2 fit within each other to fill most of the field of view. Figure 5(d) shows the cross-sectional views along the broken line A denoted in Fig. 5(c). Variant 1 and variant 2 are in contact with each other at the boundaries parallel to the [001] direction. In contrast, there are also non-contact regions between variant 1 and variant 2, where the variant boundaries are not parallel to [001]. Figure 5(e) shows a magnified view of region B shown in Fig. 5(c). Region B has ample space that is not occupied by variant 1 or variant 2, and the non-contact variant boundaries are again not parallel to [001]. Other observations in the early stage of Ni_4_Mo domain growth revealed that six orientation variants coexist in the Ni_4_Mo ordered region [100]. According to the observation result, another variant of Ni_4_Mo or possibly a Ni disordered solid solution phase occupies the empty spaces between variant 1 and variant 2 in Fig. 5(c–e). Although the specimen thickness measured from the 3D reconstructed volumes is about 50 nm, the DF-TEM images in Fig. 5(b) do not exhibit significant extinction of the image intensity inside the Ni_4_Mo domains up to the high specimen-tilt angles. This fact supports the feasibility of the 3D Ni_4_Mo domain morphology characterization described above.

Because the structure factors for superlattice reflections are generally smaller than those of fundamental lattice reflections, it is expected that the visualization of superlattice domain structures by electron back scattering diffraction (EBSD) or electron channeling contrast imaging (ECCI) in SEM is difficult. Therefore, the diffraction contrast ET demonstrated above is notably unique among various 3D electron microscopy imaging methods.

#### Diffraction contrast ET applied to dislocations

**Fig. 6:**
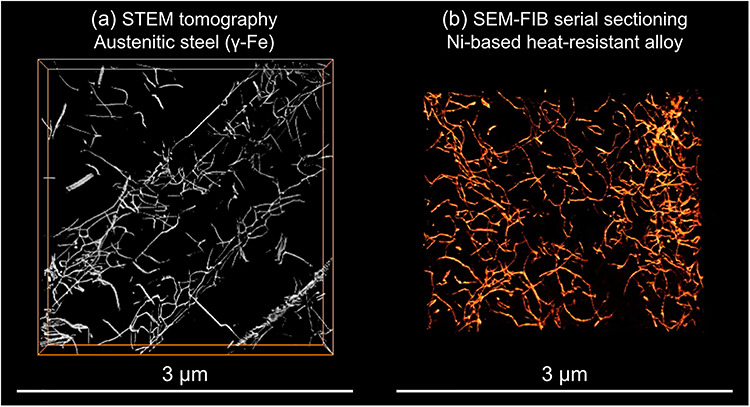
Bird’s-eye views of 3D reconstructed dislocations using different 3D electron microscopy techniques. (a) STEM tomography for a deformed austenitic steel (γ-Fe) specimen [59], and (b) SEM/FIB serial sectioning for a crept Ni-base heat-resistant alloy specimen [85].

**Fig. 7:**
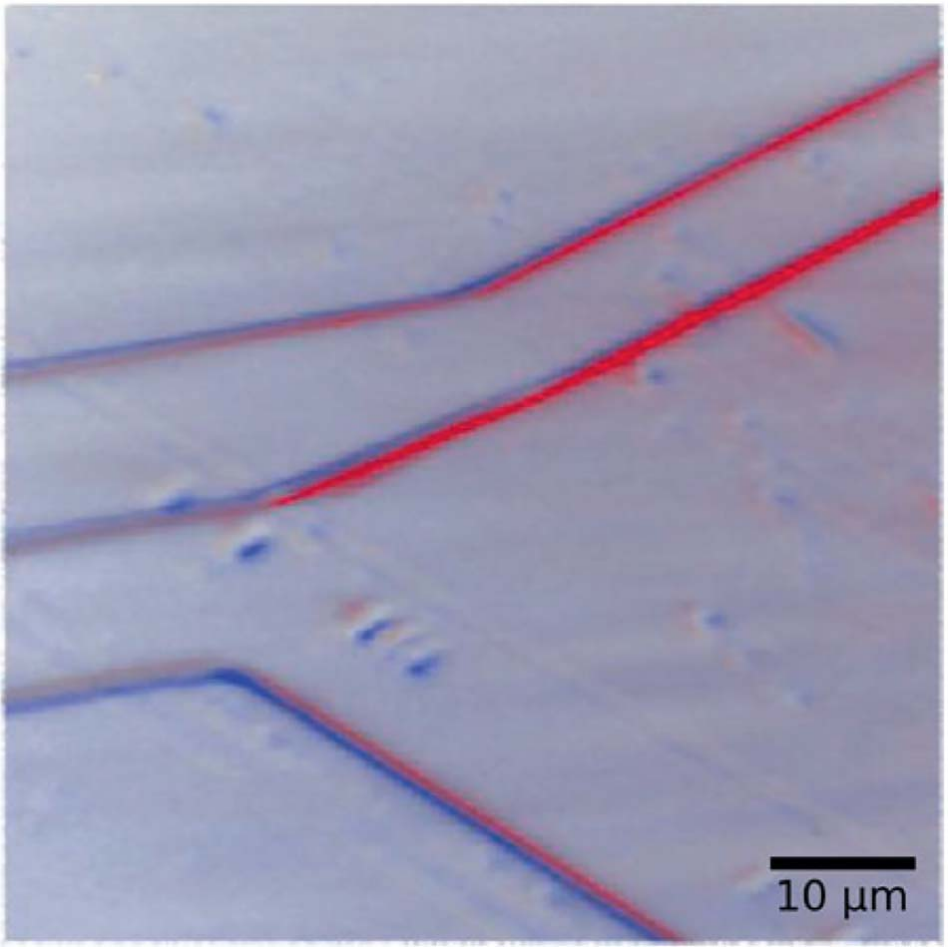
A weak-beam dark-field (WBDF) 3D X-ray microscopy image of dislocations within a diamond crystal [87]. Two 3D reconstructed volumes are superimposed: the red and blue images represent an offset in the axial strain of +3 × 10^−4^ and − 3 × 10^−4^, respectively. (The figure was reproduced under a copyright permission from Marketplace™.)

When we perform 3D imaging of dislocations, diffraction contrast ET is not a unique solution because not only TEM/STEM but also SEM and X-ray microscopy can visualize the dislocations using the diffraction phenomena in crystals. The high spatial resolution of the dislocation line contrast in SEM, TEM and STEM is suitable for imaging high-density dislocations and their substructures in metals. Recently, in the field of materials science, there has been an increase in the number of applications of SEM-ECCI to dislocations, and SEM-ECCI combined with a slice-and-view method using a FIB technique achieved 3D dislocation imaging as demonstrated in Fig. 6(b) [59, 85]. Recent state-of-the-art synchrotron X-ray microscopy is also promising for dislocation imaging. A tomographic DF transmission X-ray microscopy method that utilizes similar optics as that of DF-TEM was used to visualize 3D tensile/compressive strain fields of dislocations within a diamond crystal (Fig. 7 [87]). The 3D X-ray microscopy method achieved spatial and angular resolutions of 100 nm and 0.001°, respectively [87, 91]. The visualization of 3D strain fields in a crystal with a higher spatial resolution than X-ray microscopy would be a challenging application for ET in the future.

**Fig. 8:**
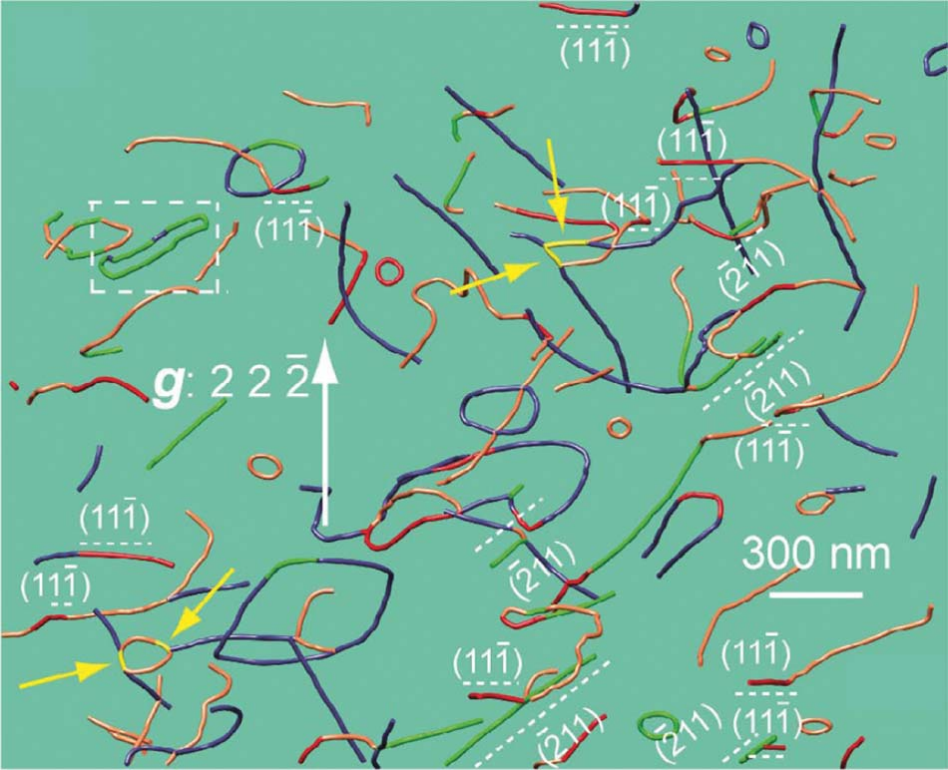
A WBDF-TEM tomography image of dislocations within an olivine (PoEM8) crystal [104]. Dislocation segments, which lie on the {111} and {211} planes, are colored in red and green, respectively, and the (11$\overline{1}$) and ($\overline{2}$11) planes are edge-on with this projection condition. The white dashed square points out a break-up of a dislocation dipole by climb, and four yellow arrows point out [10$\overline{1}$] junctions formed by dislocation climb motions. (The figure was reproduced from the open-access article [104] of Taylor & Francis Group.)

**Fig. 9:**
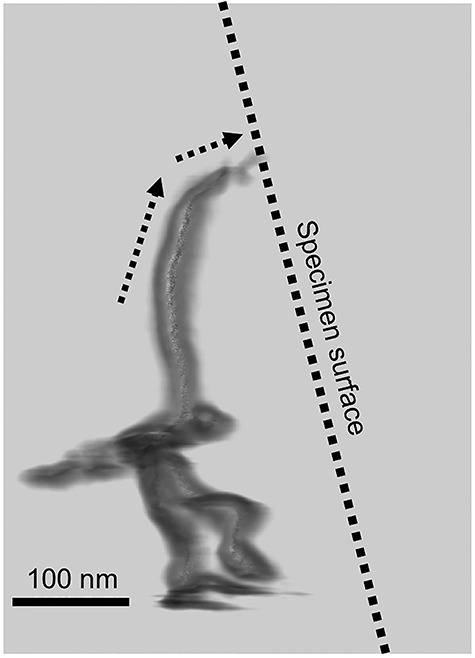
A 3D reconstructed view of a dislocation near a surface of Mo (001) thin foil [110]. The dislocation line is bent toward the free surface.

Previous reports on dislocations in foil specimens observed by high-voltage electron microscopy (HVEM) indicated that the dislocation density and morphology near a specimen surface are not the same as those in a bulk crystal [103]. Nevertheless, up-to-date observation of dislocations in a foil specimen is still useful for understanding the plasticity of crystalline materials and minerals. Furthermore, although a two-dimensional observation from different crystallographic orientations gives 3D information of the dislocations in a crystal, 3D imaging of dislocations is in demand due to various objectives, such as dislocation networks in crystalline compounds [57, 71, 96, 104 (Fig. 8)], dislocation behaviors at grain boundaries and cracks [55, 105], dislocation-precipitate interactions [106, 107], critical resolved share stress evaluated from dislocation substructures [108], FIB damage on crystal surfaces [58, 109] and influences of mirror forces on dislocation morphology near crystal surfaces (Fig. 9 [110]).

**Fig. 10:**
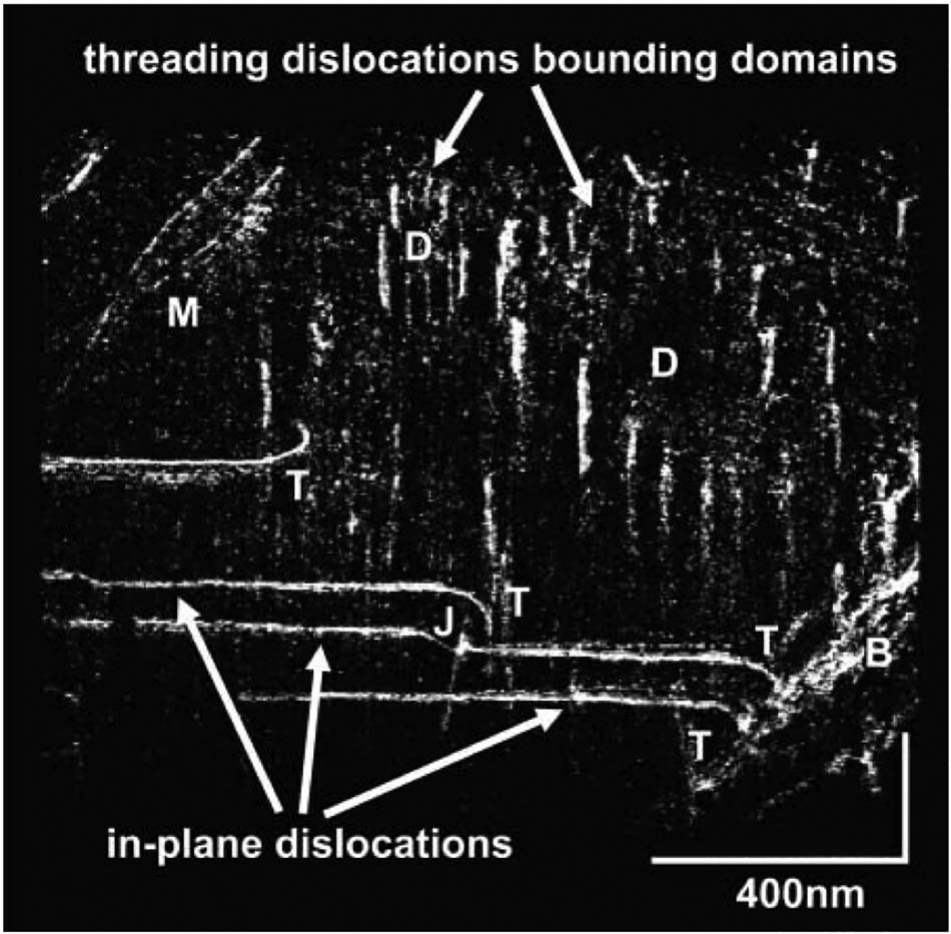
An oblique view of a WBDF-TEM tomogram of a GaN film showing walls of threading dislocations surrounding domains (D), a dislocation bundle (B) associated with a crack, and threading dislocations that turn over at T to become in-plane dislocations and terminate at the specimen surface [96]. Each turnover T occurs at a different height in the film, and one has interacted with a threading dislocation, causing a jog (J). Dislocations of mixed character (M) are also visible (this figure was reproduced under copyright permission from Marketplace™).

**Fig. 11:**
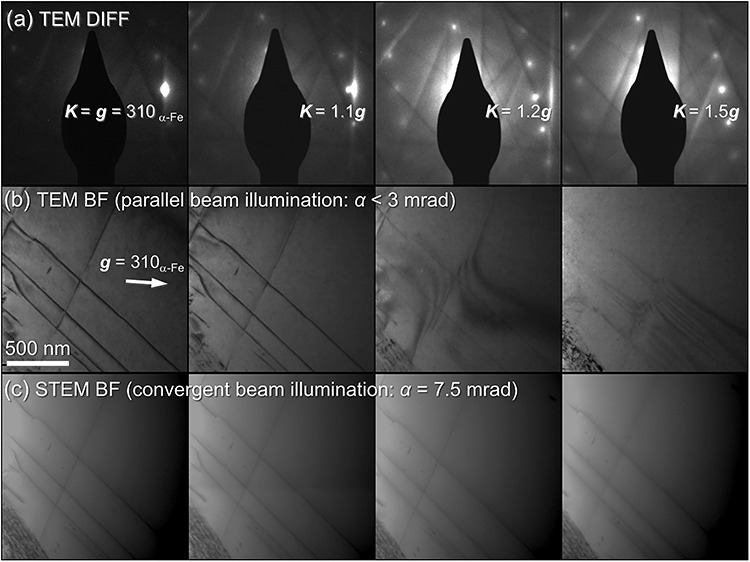
Comparison of the dependence of dislocation contrast in iron (α-Fe) on diffraction conditions between TEM (beam convergence semi-angle *α* < 3 mrad) and STEM (*α* = 7.5 mrad = 6.0 nm^−1^) [111]. (a) Selected area diffraction patterns under different diffraction conditions for ***g*** = 310_α-Fe_ (diffraction angle: 13.9 mrad = 11.1 nm^−1^ at an acceleration voltage of 200 kV). The diffraction condition is shifted from the two-beam excitation condition, ***K*** = ***g***, to the off-Bragg conditions with positive excitation errors, ***K*** = 1.1 ***g***, 1.2 ***g***, and 1.5 ***g***. (b) Corresponding BF-TEM images under the diffraction conditions in (a). The dislocation contrast becomes invisible for large excitation errors. (c) BF-STEM images under the same orientation relationships between the incident beam and the specimen as those in (b). The dislocation contrast in STEM using the convergent beam is less sensitive to the diffraction conditions than that in TEM shown in (b).

**Fig. 12:**
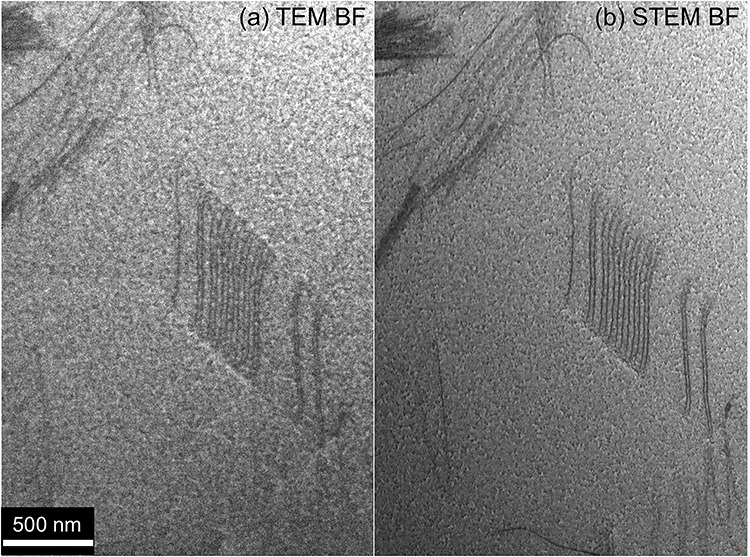
Comparison of spatial resolution of dislocation contrast in an austenitic steel iron (γ-Fe) specimen (with ~ 800 nm thickness) between (a) a BF-TEM mode (beam convergence semi-angle *α* < 3 mrad) and (b) a BF-STEM mode (*α* = 7.5 mrad = 6.0 nm^−1^) at an acceleration voltage of 200 kV [112]. The BF-STEM image in (b) shows better contrast of the dislocations than the BF-TEM image in (a).

There have been reports on suitable dislocation imaging methods for ET. Barnard *et al.* (Fig. 10 [96]), who reported ET observation of dislocations for the first time, applied a weak-beam (WB) DF-TEM to the tilt-series data set acquisition of a dislocation network in a GaN film. The WBDF-TEM tomography by Barnard *et al.* [71, 96] needed image processing to eliminate the thickness fringes that degraded the visibility of the dislocation contrast. The same research group [54, 57] and some other research groups [55, 58, 59, 107] then applied STEM instead of TEM to dislocation tomography in which the diffracted waves in the zeroth-order Laue zone contributed mainly to the STEM imaging. The STEM using a convergent incident beam weakens the dynamical diffraction contrast, such as the thickness fringes and bend contours. As a result, the visibility of dislocations in the tilt-series data set is significantly enhanced in comparison with the case of conventional TEM using a parallel beam illumination. The convergent beam illumination in STEM also widens the crystallographic orientation range in which the dislocation contrast is kept visible, as shown in Fig. 11 [111]. This feature of STEM dislocation imaging is advantageous for heavily deformed crystals, the thin foil specimens of which exhibit local variations in the crystallographic orientations for each grain [56]. Mussi *et al.* [104, 108] reported that the precession illumination in TEM provides features of dislocation contrast similar to those of the STEM dislocation contrast described above. The STEM diffraction contrast imaging is less influenced by chromatic aberration than TEM using an image formation lens system, as demonstrated in Fig. 12 [112], which is also a significant advantage of STEM diffraction contrast imaging for ET.

**Fig. 13:**
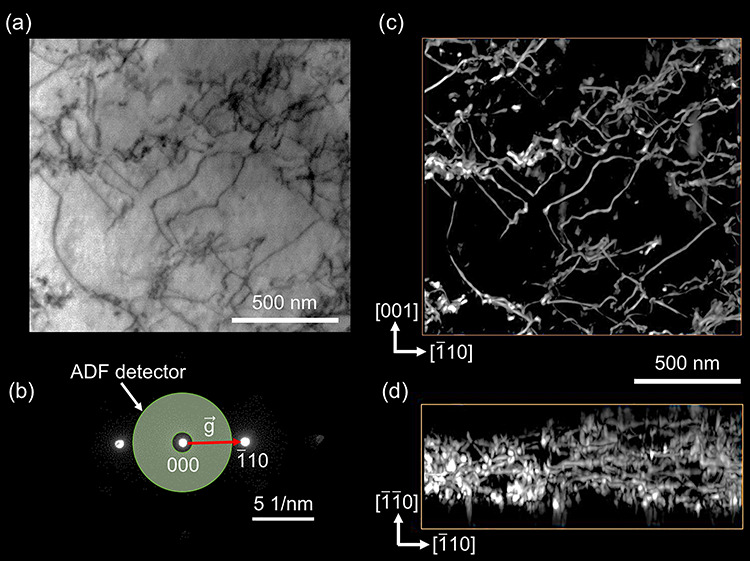
LAADF-STEM dislocation tomography for an α-Fe single-crystalline specimen with a foil normal to the (110) plane [64]. (a) The LAADF-STEM image at a specimen-tilt angle of 0°. The dislocations are visualized as dark lines. (b) The corresponding diffraction pattern and the location of the ADF detector for the LAADF-STEM imaging. Inelastically scattered electrons between the direct beam (000) and the diffracted beam ($\overline{1}10$) were detected by the ADF detector under the Bragg condition, ***K*** = ***g***($\overline{1}10$). (c) and (d) The projection views of the 3D reconstructed volume along [110] and [001], respectively. Large portions of the dislocation lines seem to lie parallel to the (110) plane since the {110} planes are dominant slip planes in the body-centered cubic α-Fe crystal. From the [001] projection in (d), the specimen thickness in the field of view is evaluated to be 300–400 nm.

As for STEM, research on the suitable imaging mode for dislocation tomography is currently in progress. The WBDF mode in which a diffracted beam is collected on a STEM detector is useful for high spatial resolution STEM imaging of dislocations [62, 63]. However, when we apply the WBDF mode to STEM dislocation tomography for a foil specimen, the image intensity of the dislocations decreases significantly at high specimen-tilt angles. Low WBDF image intensities at high specimen-tilt angles degrade the contribution of the acquired images at high specimen-tilt angles to the 3D tomographic reconstruction of dislocations. The bright-field (BF) imaging of dislocations in STEM usually shows worse spatial resolution than WBDF-STEM imaging, while the degradation of the BF dislocation image intensities at high specimen-tilt angles is less than that of the corresponding ADF dislocation image intensities under the same diffraction condition as that of BF imaging, which is advantageous for dislocation tomography [113]. Furthermore, the electron channeling contrast formed by the inelastically scattered electrons under the dynamical diffraction condition is applicable for the 3D visualization of dislocations by low-angle annular dark-field (LAADF) STEM imaging [64] as well as SEM-ECCI [85, 114, 115]. For example, the 3D visualization of dislocations in a single crystal of iron (α-Fe) thicker than 300 nm is possible with LAADF-STEM tomography using the electron channeling contrast, as shown in Fig. 13 [64]. Such findings in the image contrast of dislocations for a thick specimen are essential for ET because the electron penetration length becomes two to three times the specimen thickness during tilt-series data set acquisition. Thus, the understanding of the image formation mechanism for thick specimens is still an essential research topic [68, 116].

Because the dislocations are line defects in the crystals, there are several methods of 3D reconstruction for dislocation tomography. If we assume that the dislocation contrast is a line recognized in the matrix, a stereo pair of images is enough for the 3D reconstruction of the dislocations [61, 67, 117–120]. Nevertheless, when the dislocation density is high and/or the field of view for ET observation is large (in terms of volume), many dislocations often overlap with each other in the projection view and make 3D visualization with sufficient spatial resolution difficult. In such a case, tilt-series data set acquisition (as that performed in ET observation) helps in the selection of the best stereo pair of the projection images for 3D visualization of dislocations.

**Fig. 14:**
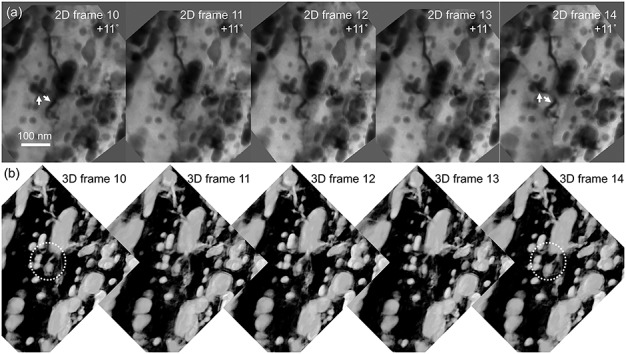
*In situ* straining and ET observation in an electropolished disc specimen of a drawn and heat-treated pearlitic steel wire. (a) 2D frames (10–14) of bright-field TEM images of the dislocations interacting with a spheroidized Fe_3_C precipitates, acquired at the same specimen-tilt angle of +11°. These images were selected from the tilt-series data sets, and small changes in the diffraction contrast (possibly dislocation contrast) are recognized, as indicated with the arrowheads. (b) Corresponding 3D frames reconstructed from the tilt-series data sets. The change in the diffraction contrast during the specimen straining contributes to the 3D reconstructed views, as denoted in the circled areas.

*In situ* 3D imaging of dislocation dynamics is a promising topic for future ET applications [117, 120, 121]. It is very challenging to observe the dislocation dynamics under a constant diffraction condition because crystal rotation, as well as crystal deformation, occurs with loading stress on the specimen for *in situ* ET observation. Therefore, diffraction alignment-free ET observation for 3D dislocation dynamics is proposed as follows: repeatedly acquire tilt-series data sets during specimen deformation without diffraction alignments; select images in which the dislocations are visible from each tilt-series data set and perform 3D reconstruction to visualize the dislocation dynamics [122]. As an exceptional example, Fig. 14 depicts a preliminary 3D observation of the dislocation dynamics in a drawn and subsequently heat-treated pearlitic Fe–C alloy specimen [123] using an *in situ* straining and ET system [41, 124]. It was revealed that some of the dislocations interacting with spheroidized Fe_3_C precipitates were visible during the sequential repetition of straining the TEM specimen and acquiring the tilt-series data [125]. The successive display of the *in situ* 2D frames acquired at the same specimen-tilt angle (+11°) and the corresponding 3D frames reconstructed from the tilt-series data are shown in Fig. 14(a) and 14(b), respectively. The small changes in diffraction contrast, denoted with arrows (Fig. 14(a)) and circles (Fig. 14(b)) suggest movement of the dislocations with the specimen straining. We found that such slight movements of the dislocations are more clearly recognized in the 3D reconstructions than in the original 2D images. This feature of the 3D imaging of dislocations will be beneficial for detailed analyses of dynamical dislocation behavior, for example, in body-centered cubic (bcc) metals which have many slip systems and often show a cross slip of screw dislocations.

**Fig. 15:**
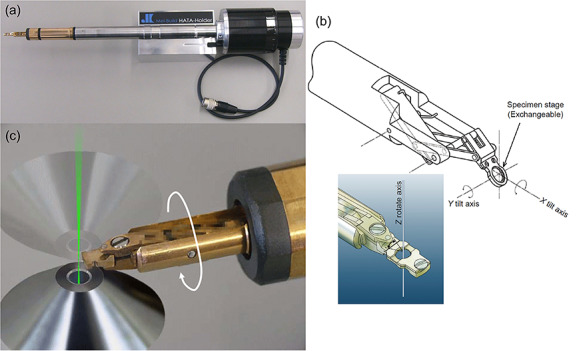
Tri-axial specimen holder developed for diffraction contrast electron tomography [59]. (a) A bird’s-eye view of the holder designed for JEOL microscopes. (b) The virtual pivot mechanism for X-Y double-tilt and Z rotation that maintains a high-angle specimen tilt along the X-tilt axis. (c) An enlarged view around the specimen stage. A needle-type specimen stage is attached to the virtual pivot system and is capable of 360° rotation and subsequent image acquisition in the HR-type pole piece of a JEOL microscope.

#### Development of experimental apparatuses for diffraction contrast ET

In principle, diffraction contrast ET requires the constant excitation of a particular diffracted wave during the tilt-series data set acquisition. One major disadvantage with using a conventional single-tilt tomography holder for diffraction contrast ET is the need for diffraction alignment by careful specimen preparation and/or specimen setting on the holder stage, which is usually a laborious and time-consuming task [95]. There is a strong demand for developing a tomography holder that performs the functions of double-tilt as well as stage-rotation, as shown in Fig. 15 [59]. Tri-axial (high-angle triple-axis (HATA)) tomography holders are capable of specimen-tilt by more than ±60° along the principal X-tilt axis, ~±7° along the secondary orthogonal Y-tilt axis and ~±5° on the perpendicular Z-rotation axis, although these angular ranges depend on the gap width and peripheral design of the objective pole piece of each electron microscope.

For the holder used for JEOL microscopes with ‘HR’-type pole pieces (Fig. 15), the wide diameter of the side-entry type holder is capable of a 360° tilt along the X-tilt axis without rotating the stage goniometer of the microscope. This 360° tilt function combined with the needle-shaped specimen preparation by FIB can be a solution for missing wedge artifacts as well as diffraction alignments. Furthermore, the free space around a specimen on the tri-axial tomography holder gives remarkably high efficiency in X-ray measurements [126]. We believe that the development of the next-generation TEMs requires further advancements in the functionality of the specimen stage system. For example, with regard to the development of fast ET data acquisition, how precisely the microscope can keep the eucentric position for the field of view during rapid specimen-tilt is a critical question. Currently, unsatisfactory movement of the specimen stage goniometer is a bottleneck in the eucentric position issue for fast ET data acquisition [42, 43].

From an MSE point of view, the development of ET imaging methods applicable for materials with magnetism, such as advanced steels and magnets, is a challenge for the future. As for imaging techniques, tomographic electron holography [92–94] and tomographic Lorentz microscopy [127] have already been developed. Recently, Hasezaki *et al.* [64] proposed a tomographic diffraction contrast STEM imaging method under a magnetic-field-free condition, which could achieve a resolution power of 5 nm using a spherical aberration corrector and demonstrated 3D visualization of dislocations in ferrous iron (α-Fe). More recently, Shibata *et al.* [128] developed a novel objective lens system for magnetic-field-free atomic-resolution TEM/STEM. Therefore, atomic-resolution ET imaging of magnetic materials may be possible in the future.

## Concluding remarks

The current status and issues of intermediate-resolution (non-atomic-resolution) ET for materials research were discussed with a particular focus on diffraction contrast ET of crystalline materials. It should be noted that other 3D imaging methods using SEM or X-ray microscopy have demonstrated promising resolution powers and functionalities, which are sometimes in competition with those of ET. Nevertheless, the merits of selecting intermediate-resolution ET as a 3D visualization method include not only the superior resolution power of TEM/STEM but also unique applications, such as the visualization of electromagnetic fields, domain structures in compound crystals and dislocation substructures in metallic materials, among others. Novel 3D reconstruction algorithms that are robust against a small number of projection image data sets and low-quality images, as well as new image recording systems suitable for rapid image acquisition under low-dose conditions, will further develop the intermediate-resolution ET imaging methods and their applications, for example, high-speed ET data set acquisition indispensable for *in situ* observations of dynamic material behaviors, such as the dynamic dislocation tomography demonstrated in this paper. In contrast, for the 3D observation of static objects that can be visualized using simple mass-thickness contrast and/or chemical composition contrast, there is a possibility that other 3D imaging methods, such as those using SEM-ECCI or X-ray microscopy, are more suitable than ET. Therefore, in the future, the selection of a suitable microscopy method will be essential for performing productive and successful 3D nanostructural analysis in materials research.

## Supplementary Material

suppl_data_dfaa002
